# Climatic factors influencing dengue incidence in an epidemic area of Nepal

**DOI:** 10.1186/s13104-019-4185-4

**Published:** 2019-03-13

**Authors:** Reshma Tuladhar, Anjana Singh, Ajit Varma, Devendra Kumar Choudhary

**Affiliations:** 1 Central Department of Microbiology, Tribhuvan University, Kathmandu, Nepal; 2 Amity Institute of Microbial Technology, Amity University, Noida, UP India

**Keywords:** Dengue, Negative binomial model, Minimum temperature, Maximum temperature, Rainfall, Relative humidity

## Abstract

**Objective:**

Geographic expansion of dengue incidence has drawn a global interest to identify the influential factors that instigate the spread of this disease. The objective of this study was to find the environmental factors linked to dengue incidence in a dengue epidemic area of Nepal by negative binomial models using climatic factors from 2010 to 2017.

**Results:**

Minimum temperature at lag 2 months, maximum temperature and relative humidity without lag period significantly affected dengue incidence. Rainfall was not associated with dengue incidence in Chitwan district of Nepal. The incident rate ratio (IRR) of dengue case rise by more than 1% for every unit increase in minimum temperature at lag 2 months, maximum temperature and relative humidity, but decrease by .759% for maximum temperature at lag 3 months. Considering the effect of minimum temperature of previous months on dengue incidence, the vector control and dengue management program should be implemented at least 2 months ahead of dengue outbreak season.

**Electronic supplementary material:**

The online version of this article (10.1186/s13104-019-4185-4) contains supplementary material, which is available to authorized users.

## Introduction

Dengue, caused by Dengue virus (DENV), is the most important mosquito-borne viral disease in the world, impacting half the world population [1, 2]. It is transmitted to human by *Aedes aegypti* and *Ae*. *albopictus* mosquitoes, and is an emerging health problem in Nepal [3]. Although classically associated with tropical climates, occurrence of dengue was reported in Nepal for the first time in 2004 from Chitwan, a lowland district with sub-tropical climate [4, 5]. Subsequently, infection has spread towards north upland hilly districts with temperate climate [6, 7]. Dengue outbreaks in Nepal have occurred in a 3 year cyclical pattern, markedly in 2010, 2013, and 2016 [7]. Upon its prevalence dengue has now been endemic in multiple districts of Nepal [8]. With expansion of DENV affected territory from 9 to 30 districts, reportedly 13 million denizens residing in the lowland Terai belt are at the risk. In addition, DENV-infected patients being identified from the hilly regions in 2013 outbreak has expanded the potential risk territory beyond the lowland belt. The spread of DENV in concurrent with the evidence of stable population of primary vector *Ae. aegypti* at an altitude of 1310 m [9, 10] suggest the growing threat of dengue in the country.

Global spread of DENV has been attributed to socio-demographic and climatic factors [11, 12]. Vector lifecycle, viral replication and host-vector interaction are linked to temperature, rainfall and humidity making dengue a climate sensitive disease [13–19]. Thus, any shift in temperature to favorable condition with adequate level of precipitation can result in expansion of vector habitats and prolongation of the transmission season.

Numerous studies have been done around the world to relate dengue incidence and climatic factors [15, 20–27]. Although, a climate-based spatial risk for dengue in Nepal has been assessed [6] the temporal trend of dengue in relation to climatic factors has not been studied. Considered as a district with significant dengue incidence, a consistent monthly dengue data since the year 2010 was recorded only from Chitwan. Thus we deemed it best to identify climatic variables and the resultant lag periods associated with dengue in Chitwan. This research will provide information to strengthen dengue control strategy and serve as a model system to investigate other *Aedes*-transmitted disease such as Chikungunya which has been recently detected in Nepal.

## Main text

### Methods

This study was conducted in Chitwan district of Nepal. Geographically located between 27°41′ to 27°70′ North latitude and 84°26′ to 84°52′ East longitude, it is situated in the mid-western region of country at an elevation of 415 m above mean sea level. The daily temperatures range between 5 to 22 °C in winter and 24 to 38 °C in summer. The monsoon season spans from June till September with average of rainfall > 100 mm.

#### Data

Monthly dengue cases from 2010 to 2017 were collected from District Public Health Office, Chitwan, Nepal. Dengue cases were reported from hospital based on detection of nonstructural protein 1 (NS1) antigen and presence of anti-dengue IgM. Daily records of maximum temperature (°C), minimum temperature (°C), rainfall (mm), and relative humidity (percentage) were obtained from the Department of Hydrology and Meteorology, Nepal. Except for the rainfall, which was calculated as cumulative monthly rainfall; monthly means were calculated from daily records of the remaining climate variables.

#### Statistical analysis

Maximum temperature, minimum temperature, rainfall and relative humidity were predictor variables while dengue case was response variable. Spearman correlation was assessed between dengue cases and climate factors to identify the most influencing preceding months (lag period) on the occurrence of dengue fever. The predictor variable of 1 month earlier was considered lag1 and the month corresponding to that of dengue case was lag 0. We selected lag period of one to 3 months for preceding months.

Since dengue case was count variable, the possible distribution may be Poisson or negative binomial. However, dengue cases were over dispersed with the variance greater than mean. Further, results of Kolmogorov–Smirnov (K–S) test showed that the dengue cases did not fit to the Poisson distribution (K–S Z = 7.098, *p* < .01). Thus, negative binomial regression for dengue cases as a response variable seemed to be appropriate for modeling. The models were attempted to include some interaction terms based on the fitting criteria. The best fit model was selected and interpreted. Similarly, incidence rate ratio (IRR) between dengue case and climatic factors was assessed to consider the relative risk of dengue incidence in relation to climatic factors. The statistical analyses were conducted using SPSS Statistics software v.21.

### Results

**Fig. 1:**
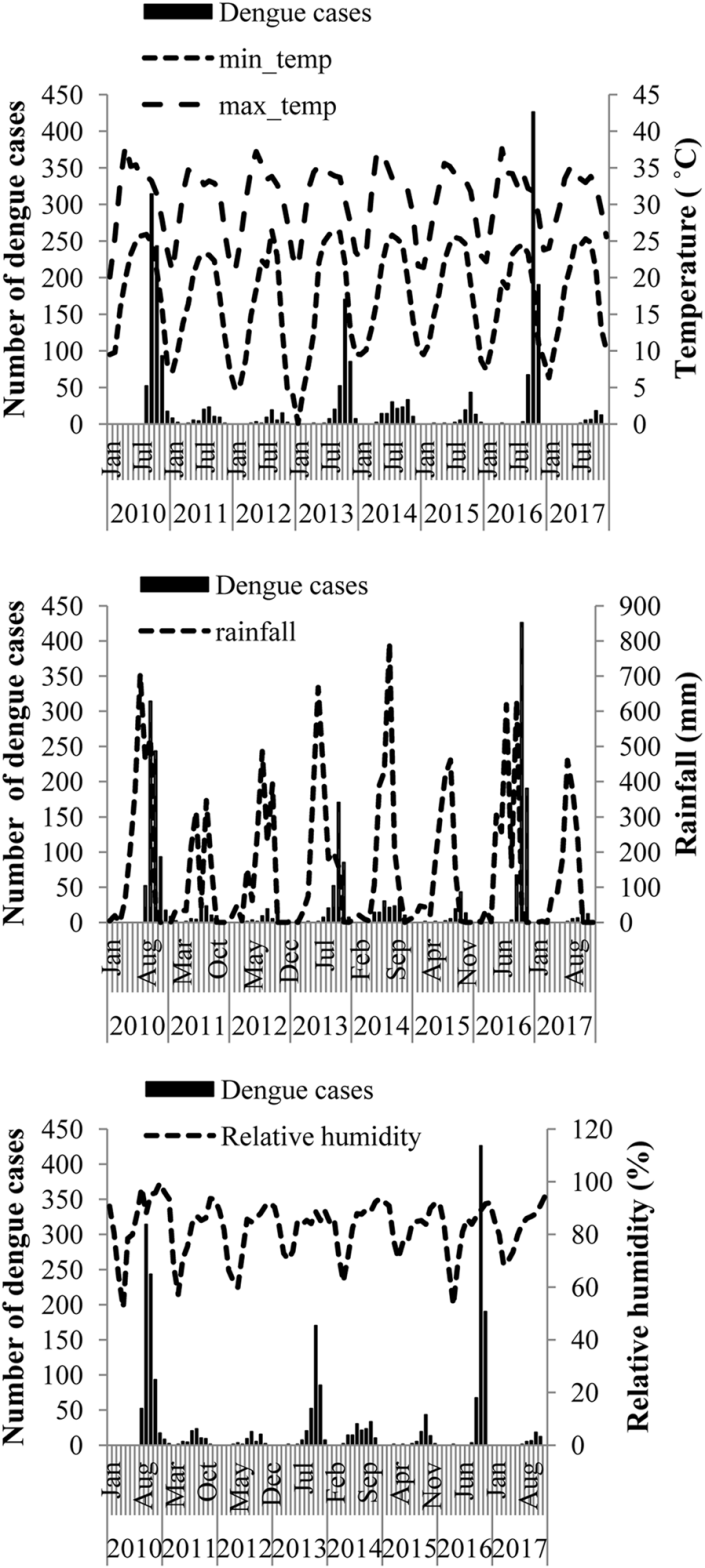
Temporal trend of monthly dengue cases against monthly climate factors from 2010 to 2017

During the 8 years period, a total of 2176 dengue cases have been reported. Yearly distribution of dengue cases showed a cyclic pattern with major outbreaks at an interval of 3 years (Additional file 1: Figure S1). A clear seasonal pattern of dengue occurrence was observed with cases progressed from July–August, hit the highest point in September–October and declined by December. The highest dengue incidence shifted from September during 2010 outbreak to October in 2013 and 2016 outbreaks (Additional file 2: Figure S2). The dengue cases reached the peak following the months with highest temperature and rainfall (Fig. 1).

**Fig. 2:**
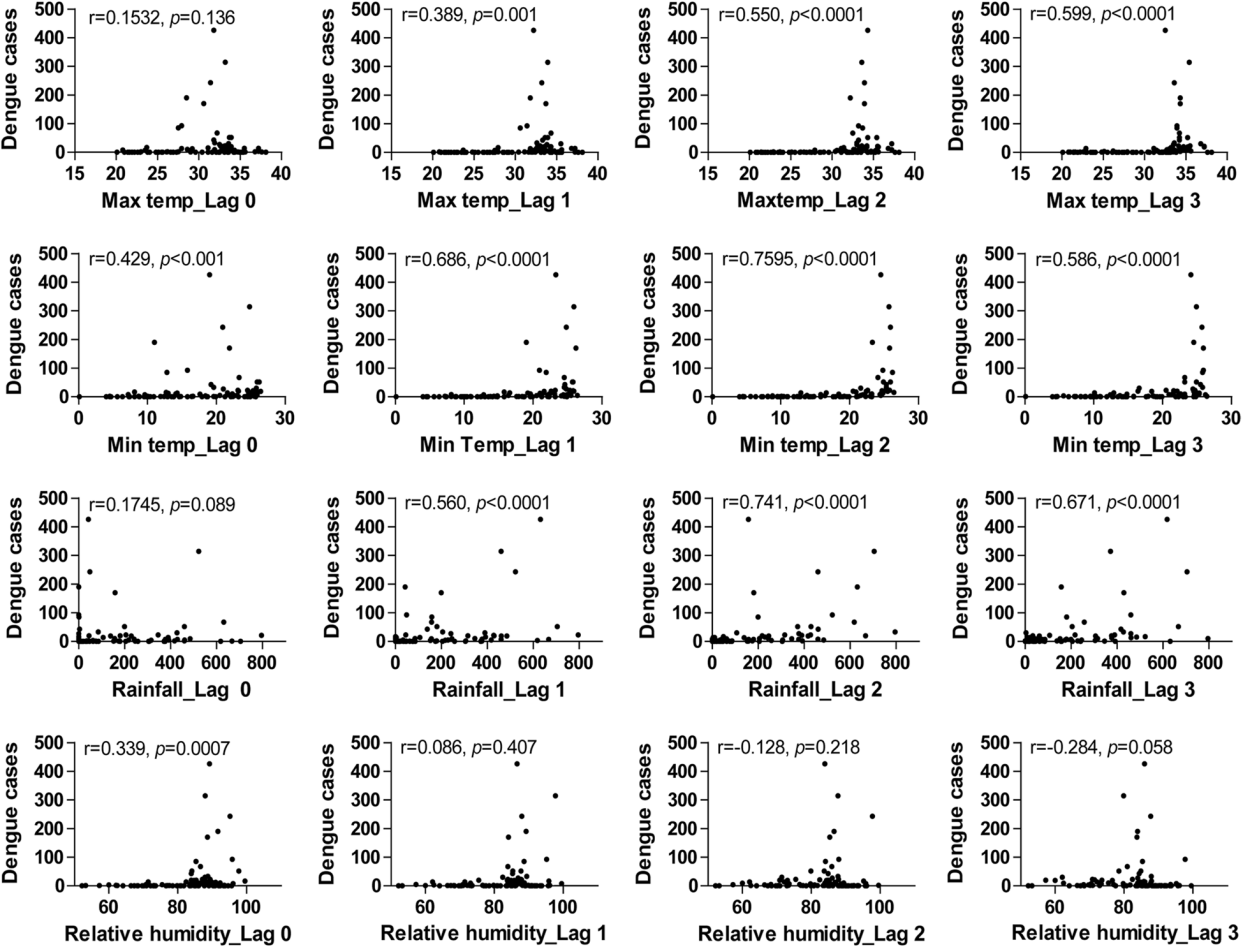
Scatterplot of relationship between climatic factors and dengue cases in Chitwan district

Spearman correlation showed temperature, rainfall and relative humidity have some significant correlation with dengue cases. Maximum temperature was significantly correlated with dengue cases through lag 1–3 month (*P* < .01) but not for the corresponding month (*P* = .136) (Fig. 2; Additional file 3: Table S1). The effect of minimum temperature was significant through lag 0–3 months with strong correlation at lag 2 (r = .7595). The strength of correlation with minimum temperature was higher compared to maximum temperature (Fig. 2). Rainfall was significantly correlated with dengue cases through lag 1–3 months with the strongest correlation at lag 2 month (r = .741) (Fig. 2). Relative humidity had moderate correlation at lag 0 month only (Fig. 2).

Negative binomial regression model was fitted for response variables on given predictor variables based on the fitting criteria. Both deviance test and omnibus test supported that seven models were well fitted to the data (Additional file 4: Table S2). The criteria of lowest value of AIC (or BIC) and mean deviance (− 2LL/df) close to one were used to select a better model. The first model included all 16 predictor variables but none were significant under Wald Chi square test (*P* < .05) (Additional file 5: Table S3). Thus, it did not meet the requirement of only significant predictors from the fitting of negative binomial regression of dengue case. Model 4 with four significant predictors (AIC = 477.15) and the model 5 with three significant predictors (AIC = 476.64) were better models.

Highly significant predictors in Model 4 under the Wald Chi square test (*P* < .05) (Table 1) were Minimum temperature at lag 2 month (Mini temp_2), Maximum temperature at lag 0 (Max temp), Maximum temperature at lag 3 month (Max temp_3) and Relative humidity at lag 0 month (Relative humidity). In order to make the interpretation simple and straight forward, partial regression coefficients were expressed in the exponential form, i.e., exp(b_i_). Exponential value greater than one indicated more effect of the predictor on the response variable and less than one indicated less effect, while one indicated no effect. Interpreting partial regression coefficients, the negative sign indicated a decrease in the response variable and the positive sign indicated an increase in the response variable per unit increase in a given predictor keeping the remaining predictors constant.

**Table 1:** Negative binomial regression model of dengue cases (Model 4)

Parameter	B	Std. error	95% Profile likelihood confidence interval		Hypothesis test			Exp(B)	95% Profile likelihood confidence interval for exp(b)	
			Lower	Upper	Wald Chi Square	df	Sig.		Lower	Upper
(Intercept)	− 10.185	4.0504	− 18.153	− 2.077	6.324	1	.012	3.771E−005	1.306E−008	.125
Mini Temp_2	.363	.0529	.258	.469	47.007	1	.000	1.437	1.295	1.598
Max Temp	.176	.0647	.048	.305	7.422	1	.006	1.193	1.049	1.356
Max Temp_3	− .275	.0882	− .454	− .101	9.747	1	.002	.759	.635	.904
Relative humidity	.104	.0411	.022	.186	6.366	1	.012	1.109	1.023	1.204
(Scale)	1^a^									
(Negative binomial)	1.628	.3167	1.120	2.412						

Dependent Variable: Dengue cases

Model: (Intercept), Mini Temp_2, Max Temp, Max Temp_3, Relative humidity

^a^Fixed at the displayed value

The variable Mini temp_2 has a coefficient of .363, which implies that for each one degree Celsius increase in minimum temperature at lag 2 month, the expected log of dengue case increases by .363. Similarly for Max temp with a coefficient of .176, one degree increase in maximum temperature of the corresponding month, the expected log of dengue cases increases by .176. Likewise, same implied for Relative humidity with value of .104 where one percentage increase in relative humidity, the expected log of dengue cases increases by .104.

For Max temp_3 which has a coefficient value in minus, for each one degree increase in maximum temperature at lag 3 month, the expected log of dengue cases decreases by .275. Min temp_2 [exp (.363) = 1.437], Max temp [exp (.176) = 1.193] and Relative humidity [exp (.104) = 1.109] have values greater than one, but Max temp_3 [exp (− .275) = .759] has value less than one. This means minimum temperature at lag 2 month, Maximum temperature and Relative humidity at 0 lag have shown greater impact on dengue incidence. Among them, minimum temperature at lag 2 month was the most influential.

Similarly, the percent change in the incident rate ratio (IRR) of dengue cases was a 1.437%, 1.193% and 1.109% increase for every unit increase in Mini temp_2, Max temp and relative humidity respectively, holding other predictors constant. However, for every unit increase in Maxtemp_3, the percent change in the IRR of dengue cases decreased by .759%.

Model 5 was not chosen since it included only three significant predictors and the regression coefficients values were less compared to model 4 (Additional file 4: Table S3).

### Discussion

In this study, important environmental determinant that influenced dengue incidence in an epidemic Chitwan District of Nepal was identified.

A persistent peak in dengue cases each year following the highest rainfall and temperature, observed in the time series graph, in conjunction with significant higher correlation of response variable with these predictor variables at lag periods (Fig. 2) indicated the influence of preceding month’s climatic factors, which was comparable to other countries [12, 28, 29]. We selected lag period of 1 to 3 months taking into consideration the time for an adult *Aedes* spp. to develop from egg, extrinsic and intrinsic incubation period of virus [30] and time for patient to visit hospital following onset of symptom.

The correlation analysis is limited to considering individual relationship and does not address the co-effect of other climate factors, thus we further performed regression analysis to elucidate the true association. Despite a significant relation between dengue cases and rainfall observed in correlation analysis, rainfall was not included as a significant predictor in the regression model. The model that included rainfall showed a low coefficient value, however this model was not selected, hence rainfall might have less influence on dengue incidence in Chitwan. Dengue transmission was not related to rainfall in numerous countries [13, 31, 32]. Influence of rainfall on dengue incidence [33, 34] has been linked to increase in vector population [35]. However, it cannot constantly be valid since excessive rainfall has negative impact [36, 37]. Vector breeding was also possible in dry season when water stored artificial containers were available [38]. *Ae. aegypti*, the major driver of dengue transmission [34, 39] usually breed in artificial containers [34]. Their abundance in Chitwan compared to *Ae*. *albopictus* [40] gave an impression that water storage behavior can also be a potential contributing factor for dengue transmission which needs to be further investigated in Chitwan.

In view of *Aedes* spp. abundance regulated by temperature than precipitation [41, 42], temperature has profound impact on dengue prevalence. Temperature strongly influenced dengue cases in several countries [20, 24, 28, 31, 43–46]. High risk of dengue identified with temperature at lag period between 1 and 3 months [20, 28, 31] was close to the minimum temperature at lag 2 months seen in our study. The minimum temperature at lag 2 months in Chitwan was between 23 and 25 °C, which was conducive for vector survival [47] compared to temperature at lag 0. Besides, higher rate of dengue virus dissemination influenced by mosquito vector at 25 °C compared to 20 °C [48] was also in favor of Min temp_2.

Amplification of dengue virus within *Ae. aegypti* was augmented at 24–31 °C [49] and the maximum temperature of the month with peak dengue incidence in Chitwan was closer to the upper limit of this range. Thus a positive effect of maximum temperature at lag zero month might be due to the influence of vectoral capacity. However, a negative relationship of maximum temperature observed at lag 3 months can be due to temperature higher than 35 °C around this month in Chitwan. The reverse effect of lag 3 months was also observed in Vietnam [29]. Influence of relative humidity on vector survival and dengue transmission in combination with temperature [50] corroborates the significant effect of maximum temperature and relative humidity in the month with lag zero.

We found dengue peak month shifted from September to October subsequently from 2013. This shift may be linked to climate change, adaptation of vector and human behavior. Though climate change is a current global concern we cannot validate its effect from 8 years study period. As the relationship between climatic factors and dengue incidence vary with countries and geographical location [15, 20, 37, 43, 51], researches to address factors owing to dengue outbreaks and geographic extension are essential for Nepal which represents a diverse climate within a short latitudinal range.

### Conclusion

This study has identified temperature and relative humidity as potential contributors, with minimum temperature at lag 2 months being the most significant, for dengue prevalence in lowland Chitwan district of Nepal. We suggest that vector control program for dengue containment will be more effective if implemented from the months of June–July.

### Limitations

Climatic factors are not the sole predictor of vector borne disease like dengue. Socio-demographic components such as population growth, travel or migration rate, water storage habit should also be considered to link with incidence of dengue. This work has been limited to single district, hence in future several districts encompassing diverse geographic region should be studied.

## Additional files

**Additional file 1: Figure S1.** Yearly distribution of dengue cases in Chitwan from 2010 to 2017.

**Additional file 2: Figure S2.** Dengue cases by month from 2010 to 2017 in Chitwan district.

**Additional file 3: Table S1.** Correlation analysis between dengue cases and climate factors with lag effects of 0–3 months period.

**Additional file 4: Table S2.** A goodness of fit test for a response variable using negative binomial regression model.

**Additional file 5: Table S3.** Negative binomial regression models of dengue cases.

## Abbreviations

AIC: Akaike’s information criterion
BIC: Bayesian information criterion
DENV: dengue virus
IRR: incident rate ratio
