# Supplementary figures and images for: Climatic factors influencing dengue incidence in an epidemic area of Nepal
Source: BMC Res Notes. 2019 Mar 13;12:131. doi: 10.1186/s13104-019-4185-4 (PMC6417253; doi:10.1186/s13104-019-4185-4)

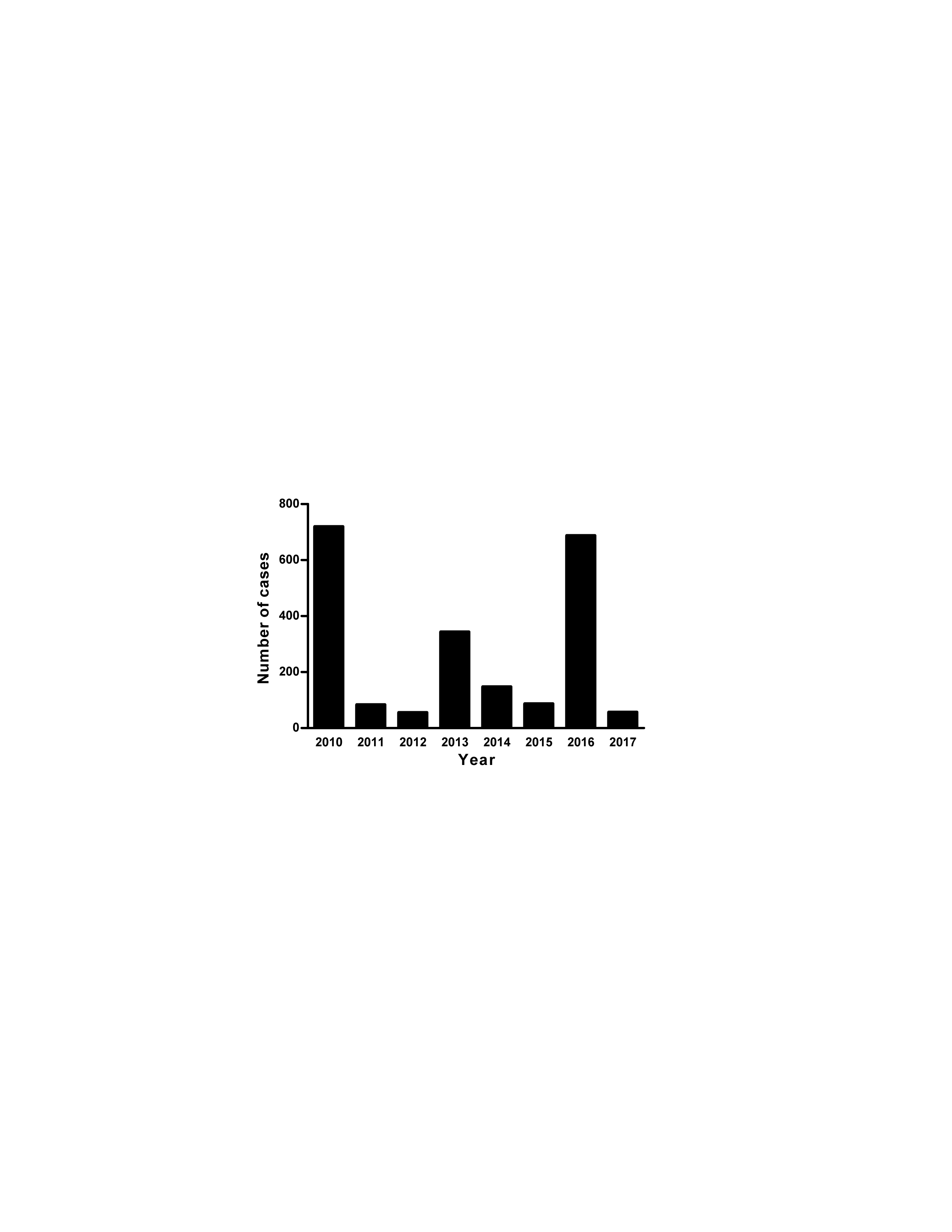

Supplement: Supplementary file 1 — Additional file 1: Figure S1. Yearly distribution of dengue cases in Chitwan from 2010 to 2017. [file 13104_2019_4185_MOESM1_ESM.tif]

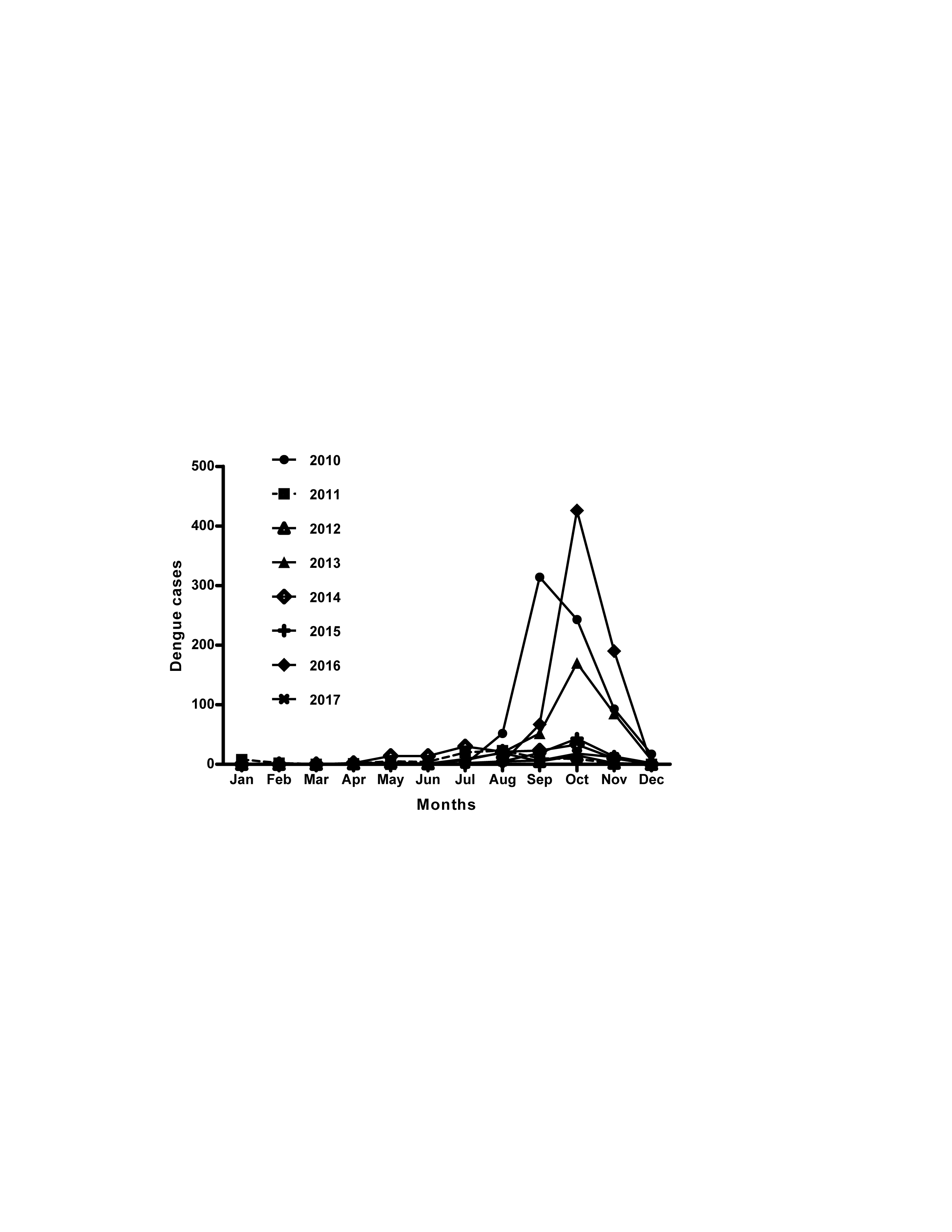

Supplement: Supplementary file 2 — Additional file 2: Figure S2. Dengue cases by month from 2010 to 2017 in Chitwan district. [file 13104_2019_4185_MOESM2_ESM.tif]
