# Supplementary material for: Climatic factors influencing dengue incidence in an epidemic area of Nepal
Source: BMC Res Notes. 2019 Mar 13;12:131. doi: 10.1186/s13104-019-4185-4 (PMC6417253; doi:10.1186/s13104-019-4185-4)
Supplement: Supplementary file 3 — Additional file 3: Table S1. Correlation analysis between dengue cases and climate factors with lag effects of 0–3 months period. [file 13104_2019_4185_MOESM3_ESM.docx]

**Additional file 3: Table S1** Correlation analysis between dengue cases and climate factors with lag effects of 0-3 months period.

| **Climate variable** | **Lag month** | **Correlation** | ***P*-value** |
| --- | --- | --- | --- |
| Maximum temperature | 0 | 0.1532 | 0.136 |
|  | 1 | 0.389^*^ | 0.001 |
|  | 2 | 0.550^**^ | <0.0001 |
|  | 3 | 0.599^**^ | <0.0001 |
| Minimum temperature | 0 | 0.429^**^ | <0.0001 |
|  | 1 | 0.686^**^ | <0.0001 |
|  | 2 | 0.7595^**^ | <0.0001 |
|  | 3 | 0.586^**^ | <0.0001 |
| Rainfall | 0 | 0.1745 | 0.089 |
|  | 1 | 0.560^**^ | <0.0001 |
|  | 2 | 0.741^**^ | <0.0001 |
|  | 3 | 0.671^**^ | <0.0001 |
| Relative humidity | 0 | 0.339 | 0.0007 |
|  | 1 | 0.0856 | 0.407 |
|  | 2 | -0.128 | 0.218 |
|  | 3 | -0.284 | 0.058 |
