# Supplementary material for: Climatic factors influencing dengue incidence in an epidemic area of Nepal
Source: BMC Res Notes. 2019 Mar 13;12:131. doi: 10.1186/s13104-019-4185-4 (PMC6417253; doi:10.1186/s13104-019-4185-4)
Supplement: Supplementary file 4 — Additional file 4: Table S2. A goodness of fit test for a response variable using negative binomial regression model. [file 13104_2019_4185_MOESM4_ESM.docx]

**Additional file 4:Table S2.** A goodness of fit test for a response variable using negative binomial regression model

| **Models** | **No. of Predictor** | **Deviance** | | **Omnibus Test** | | **AIC** | **BIC** |
| --- | --- | --- | --- | --- | --- | --- | --- |
|  |  | **-2LL** | **Df** | **Likelihood Ratio**  **χ^2^ (df)** | **Sig** |  |  |
| 1 | 16 | 78.81 | 62 | 88.38 (16) | .000 | 490.00 | 533.32 |
| 2 | 4 | 81.90 | 74 | 73.43(4) | .000 | 482.39 | 495.69 |
| 3 | 4 | 81.78 | 74 | 66.94(4) | -000 | 487.88 | 502.18 |
| 4 | 4 | 79.91 | 74 | 78.83(4) | -000 | 477.15 | 490.29 |
| 5 | 3 | 80.37 | 75 | 76.99(3) | .000 | 476.64 | 487.74 |
| 6 | 3 | 81.31 | 75 | 71.98(3) | .000 | 481.66 | 492.76 |
| 7 | 3 | 81.46 | 75 | 71.84(3) | .000 | 481.80 | 492.90 |
