# Supplementary material for: Climatic factors influencing dengue incidence in an epidemic area of Nepal
Source: BMC Res Notes. 2019 Mar 13;12:131. doi: 10.1186/s13104-019-4185-4 (PMC6417253; doi:10.1186/s13104-019-4185-4)
Supplement: Supplementary file 5 — Additional file 5: Table S3. Negative binomial regression models of dengue cases. [file 13104_2019_4185_MOESM5_ESM.docx]

**Additional file 5: Table S3.**  Negative binomial regression models of dengue cases

| **Models** | **1** | **2** | **3** | **4** | **5** | **6** | **7** |
| --- | --- | --- | --- | --- | --- | --- | --- |
| Parameter | B  (SE) | B  (SE) | B  (SE) | **B**  **(SE)** | **B**  **(SE)** | B  (SE) | B  (SE) |
| (Intercept) | -27.291^**^  (11.811) | -21.835^*^  (5.478) | -6.363^*^  (1.592) | **-10.186^**^**  **(4.050)** | **-12.947^*^**  **(2.607)** | -5.48^*^  (1.425) | -5.364^*^  (1.437) |
| Mini Temp | -0.047  (0.091) |  |  |  |  |  |  |
| Mini Temp_1 | 0.033  (0.133) | 0.162^**^  (.068) | 0.278  (.043) |  |  |  |  |
| Mini Temp_2 | 0.116  (0.176) |  |  | **0.363^*^**  **(0.0529)** | **0.324^*^**  **(0.092)** | 0.246^*^  (0.037) | 0.213^*^  (0.0385) |
| Mini Temp_3 | 0.059  (0.135) |  | -0.225^**^  (.089) |  |  |  |  |
| Max Temp | 0.201  (0.193) |  |  | **0.176^*^**  **(0.065)** | **0.142^*^**  **(0.0437)** | 0.086^**^  (0.039) |  |
| Max Temp_1 | 0.227  (0.209) | 0.352^*^  (.131) |  |  |  |  | 0.098^**^  (0.0468) |
| Max Temp_2 | 0.034  (0.231) |  |  |  |  |  |  |
| Max Temp_3 | -0.069  (.210) |  |  | **-0.275^*^**  **(0.0882)** |  |  |  |
| Rainfall | -0.002  (0.0013) | -0.004^*^  (0009) |  |  |  |  |  |
| Rainfall_1 | 0.001  (0.0014) |  |  |  |  |  |  |
| Rainfall_2 | 0.001  (0.0015) |  | -0.003^**^  (.0010) |  |  |  |  |
| Rainfall_3 | -0.001 |  |  |  |  | 0.002^***^  (0.0011) | 0.002^**^  (0.0011) |
| Relative humidity | 0.07  (0.0016) |  | 0.119^*^  (.0313) | **0.104^**^**  **(0.0411)** |  |  |  |
| Relative humidity_1 | 0.055  (0.049) | 0.128^*^  (.032) |  |  |  |  |  |
| Relative humidity _2 | -0.008  (.051) |  |  |  |  |  |  |
| Relative humidity _3 | 0.054 |  |  |  | **0.057^*^**  **(0.0174)** |  |  |
| Dispersion parameter(Alpha) | 1.4  (0.279) | 1.727  (.3366) | 1.932  (.3677) | **1.629**  **(0.3167)** | **1.667**  **(0.3237)** | 1.788  (0.3443) |  |

Note: ^*^Significant at 1%, ^**^Significant at 5%, and ^***^Significant at 10% on the basis of Wald Chi-Square

Abbreviation: SE standard error

Mini Temp_1 Minimum temperature at lag 1 month, Mini Temp_2 Minimum temperature at lag 2 months, Mini Temp_3 Minimum temperature at lag 3 month

Max Temp_1 Maximum temperature at lag 1 month, Max Temp_2 Maximum temperature at lag 2 months, Max Temp_3 Maximum temperature at lag 3 months
